# Effectiveness and Tolerability of Dual Antiviral Therapy in Immunosuppressed Patients with Protracted SARS-CoV-2 Infection

**DOI:** 10.3390/idr17020017

**Published:** 2025-02-26

**Authors:** Giovanna Travi, Francesco Peracchi, Marco Merli, Emanuele Ravano, Anna Frustaci, Marina Deodato, Diana Fanti, Alice Nava, Valeriana Colombo, Nicholas Brian Bana, Carlotta Rogati, Alessandro Raimondi, Cristina Moioli, Anna Maria Pazzi, Marta Vecchi, Davide Motta, Roberto Rossotti, Chiara Oltolini, Fulvio Crippa, Enrico Minetti, Chiara Vismara, Roberto Cairoli, Massimo Puoti

**Affiliations:** 1 Division of Infectious Diseases, ASST Grande Ospedale Metropolitano Niguarda, Piazza dell’Ospedale Maggiore, 3, 20162 Milan, Italy; giovanna.travi@ospedaleniguarda.it (G.T.); marco.merli@ospedaleniguarda.it (M.M.); n.bana@campus.unimib.it (N.B.B.); carlotta.rogati@ospedaleniguarda.it (C.R.); alessandro.raimondi@ospedaleniguarda.it (A.R.); mariacristina.moioli@ospedaleniguarda.it (C.M.); annamaria.pazzi@ospedaleniguarda.it (A.M.P.); marta.vecchi@ospedaleniguarda.it (M.V.); davide.motta@ospedaleniguarda.it (D.M.); roberto.rossotti@ospedaleniguarda.it (R.R.); chiara.oltolini@ospedaleniguarda.it (C.O.); fulvio.crippa@ospedaleniguarda.it (F.C.); massimo.puoti@ospedaleniguarda.it (M.P.); 2 School of Medicine and Surgery, University of Milano-Bicocca, 20162 Monza, Italy; 3 Division of Hematology, ASST Grande Ospedale Metropolitano Niguarda, 20162 Milan, Italy; emanuele.ravano@ospedaleniguarda.it (E.R.); annamaria.frustaci@ospedaleniguarda.it (A.F.); marina.deodato@ospedaleniguarda.it (M.D.); roberto.cairoli@ospedaleniguarda.it (R.C.); 4 Division of Clinical Microbiology, ASST Grande Ospedale Metropolitano Niguarda, 20162 Milan, Italy; diana.fanti@ospedaleniguarda.it (D.F.); alice.nava@ospedaleniguarda.it (A.N.); chiara.vismara@ospedaleniguarda.it (C.V.); 5 Division of Nephrology, ASST Grande Ospedale Metropolitano Niguarda, 20162 Milan, Italy; valeriana.colombo@ospedaleniguarda.it (V.C.); enrico.minetti@ospedaleniguarda.it (E.M.)

**Keywords:** dual antiviral therapy, SARS-CoV-2, immunodepression, hematological, SOT

## Abstract

Background: Immunosuppressed patients still exhibit a high mortality rate due to SARS-CoV-2 infection, up to 21%. Persistent viral load replication and protracted viral symptoms result in a high risk of developing pneumonia, a potential risk of antiviral resistance, and a subsequent delay of onco-hematological treatments. Methods: Hematological patients and kidney transplant patients with SARS-CoV-2 infection, treated at GOM Niguarda Hospital (Milan) with combined antiviral therapy (remdesivir plus nirmatrelvir/ritonavir at standard doses) between November 2022 and March 2024, were retrospectively reviewed. Results: Thirty-four patients were analyzed. Twenty-four (71%) patients had pneumonia. The median duration of SARS-CoV-2 positivity before antiviral treatment was 40 (10–34) days. The median treatment duration was 11 (10–10) days. All patients went through clinical resolution. Thirteen patients were exposed to a new immune-chemotherapy cycle early after antiviral treatment (median 13, IQR 6–12 days), while five resumed a standard immunosuppressive regimen immediately after viral clearance. No relapse or recurrence of symptoms was reported for up to 226 (106–318) days of follow-up. Antiviral therapy was well tolerated, and no adverse events were observed. The 30-day overall survival was 94%, while the 90-day survival was 88%. No patient died of SARS-CoV-2 infection. Conclusions: The administration of nirmatrelvir/ritonavir and remdesivir lead to the complete resolution of SARS-CoV-2 pneumonia with no side effects in this cohort. The combination of these two antivirals may be a safe option in immunosuppressed population at risk of severe complications and prolonged SARS-CoV-2 infection in order to treat severe clinical presentation and to avoid viral recurrence after chemotherapy.

## 1. Introduction

Protracted SARS-CoV-2 infection, characterized by persistent viral positivity, recurring symptoms, and radiological signs lasting more than 21 days [1], presents a significant challenge in managing hematological patients. In this population, the clinical course tends to be more severe, with higher mortality rates compared to the general population. Furthermore, short courses of antiviral therapy often fail to achieve viral clearance [2,3,4].

Viral infections in immunocompromised individuals are typically marked by higher viral loads and extended viral replication, increasing the risk of immune-escape variants with complex resistance patterns to antiviral treatments and monoclonal antibodies [5]. Recent clinical data have highlighted these populations, where prolonged SARS-CoV-2 nasal swab positivity leads to a heightened risk of disease recrudescence and severe complications, such as recurrent pneumonia and respiratory failure [1,6]. Notably, even with Omicron subvariants, hematological patients experience significantly higher mortality due to prolonged viral shedding, protracted illness, and severe disease presentations compared to immunocompetent individuals [7]. Additionally, patients treated with chemotherapy within the past three months—and especially within two weeks of SARS-CoV-2 infection—face increased mortality [8]. As a result, current guidelines recommend confirming both clinical and virological resolution of COVID-19 before resuming chemotherapy [8]. Emerging evidence supports the safety of proceeding with Chimeric Antigen Receptor T-cell (CAR-T) therapy and hematopoietic stem cell transplantation (HSCT) after viral clearance [9].

Solid organ transplant (SOT) recipients also exhibit higher morbidity and mortality from SARS-CoV-2 infection compared to the general population [10,11,12]. However, unlike hematological patients, SOT recipients generally experience lower rates of severe and prolonged infection, as immunosuppressive therapy can often be reduced or withdrawn to facilitate viral clearance [13,14].

Both nirmatrelvir/ritonavir and remdesivir are approved for treating SARS-CoV-2 infection in the general and immunocompromised populations without dosage or indication differences [7]. Remdesivir, a nucleotide analog prodrug, is metabolized intracellularly to its active form, remdesivir triphosphate, which competes with adenosine triphosphate (ATP) for incorporation into viral RNA, leading to delayed chain termination during RNA replication [15]. Nirmatrelvir, a peptidomimetic inhibitor of the SARS-CoV-2 main protease (also known as 3C-like protease or nsp5 protease), prevents viral replication by blocking polyprotein processing. Ritonavir, in turn, inhibits the CYP3A-mediated metabolism of nirmatrelvir, thereby increasing its plasma concentration [16].

Current evidence indicates that single, short-course antiviral regimens are ineffective in treating vulnerable populations such as immunosuppressed patients [2,3,4], and combination therapies have not yet been approved. This report explores the use of a combined antiviral regimen with remdesivir plus nirmatrelvir/ritonavir in hematological patients and kidney transplant recipients with SARS-CoV-2 infection.

## 2. Methods

A retrospective analysis was conducted on 34 consecutive patients treated with combined antiviral therapy comprising remdesivir (200 mg on day one, followed by 100 mg daily) and nirmatrelvir/ritonavir (100 mg ritonavir BID plus 1 or 2 nirmatrelvir BID, depending on renal function) between November 2022 and March 2024. The combination therapy was used off-label. This monocenter study was performed at ASST Grande Ospedale Metropolitano Niguarda in Milan.

Data from the first six patients were previously reported [17].

Blood tests, including complete blood count, C-reactive protein, procalcitonin, aspartate and alanine aminotransferase, creatinine, urea, and sodium and potassium levels, were performed at least twice weekly, while clinical parameters were recorded daily. Liver and kidney function, as well as renal clearance, was frequently monitored to assess potential adverse effects from the antiviral therapies.

Drug dosages were adjusted based on renal function according to drug data sheets, and concomitant medications were modified or discontinued to minimize drug–drug interactions. Plasma concentrations of calcineurin inhibitors were monitored daily and adjusted as needed to maintain therapeutic levels. Electrocardiograms were conducted upon admission and subsequently on a weekly basis.

SARS-CoV-2 detection was carried out using RT-PCR on nasopharyngeal swabs (STANDARD M10™ SARSCOV-2, SD BIOSENSOR, Sejong, Republic of Korea), bronchoalveolar lavage (BAL) fluid (Xpert^®^ Xpress COV-2 Plus, Cepheid, Sunnyvale, CA, USA), or antigen tests (STANDARD Q COVID-19 Ag, SD BIOSENSOR, Republic of Korea) from nasal swabs. SARS-CoV-2 serological testing (CMIA for SARS-CoV-2 IgG anti-N and CMIA for SARS-CoV-2 IgG II Quant for RBD, Abbott, Chicago, IL, USA) and interferon-gamma release assays (covi-FERON FIA, SD BIOSENSOR, Republic of Korea) were performed at the time of infection.

Antigen testing was performed on days 5 and 10 of treatment. RT-PCR on nasopharyngeal swabs was used to confirm viral clearance. In cases where RT-PCR was unavailable, two consecutive negative antigen tests, performed 24 h apart, were required to define a virological cure.

Treatment continued until both clinical cures, defined as the resolution of COVID-19 symptoms, and virological clearance were achieved.

All 34 patients underwent antigen nasal swab testing on days 5 and 10, and 27 also had RT-PCR confirmation on day 10. Three patients underwent repeated antigen testing and RT-PCR after 15, 20, and 25 days. Four patients were initially positive based on BAL samples, and only one patient underwent repeat bronchoscopy. Patient survival was calculated using the Kaplan–Meier method; the curves obtained were compared using the log-rank test.

Data management and analysis were performed using the MEDcalc software package, version 19.6.1.

## 3. Results

Thirty-four patients were analyzed. All patients received three or four doses of the mRNA SARS-CoV-2 vaccine; the last dose was administrated in the last six months before the infection. However, 12 patients (among 19 testes) had a negative cell-mediated immune response.

All subjects were infected by Omicron subvariants. Eighteen (53%) patients had already been treated with antiviral therapy at the onset of symptoms with remdesivir or nirmatrelvir/ritonavir (respectively, thirteen and five patients). Twenty-four (71%) patients had pneumonia; SARS-CoV-2 was isolated only through bronchoalveolar lavage in four subjects, nine (26%) patients experienced just rhinitis or fever, and one patient was asymptomatic. The median duration of SARS-CoV-2 positivity before antiviral treatment was 40 (10–34) days. The median treatment duration was 11 (10–10) days. All patients went through clinical resolution. Twenty-seven patients (80%) achieved a negative antigen nasal swab within 10 days; among them, twenty-four also had concomitantly confirmed viral clearance by RT-PCR. The other three patients (11%) achieved antigen test and RT-PCR negativity with longer treatment times of, respectively, 20, 25, and 15 days. Radiological resolution was documented in all four patients with just BAL positivity. Just one patient underwent a subsequent BAL, confirming SARS-CoV-2 undetectability on day 27. All clinical characteristics are presented in Table 1.

Thirteen patients were exposed to a new immune-chemotherapy cycle (rituximab was administered in 4/13 cases) early after antiviral treatment (median 13 [6,7,8,9,10,11,12] days), while five patients resumed a standard immunosuppressive regimen (calcineurin inhibitor plus mycophenolate mofetil) immediately after viral clearance. No relapse or recurrence of symptoms was reported for up to 226 (106–318) days of follow-up.

Antiviral therapy was well tolerated, and no adverse events were reported.

**Figure 1:**
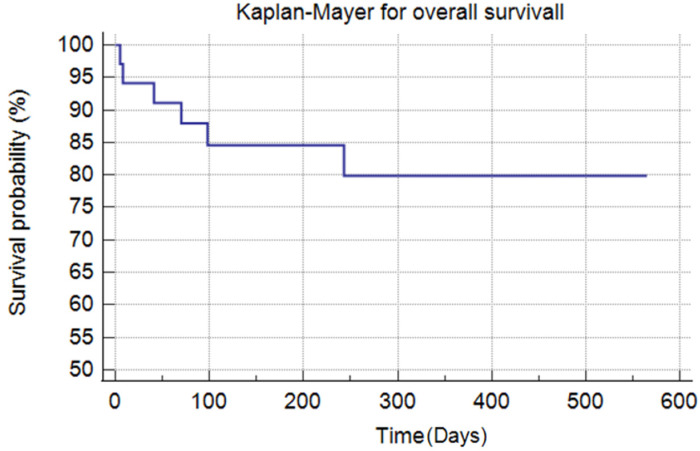
Kaplan–Mayer for overall survival.

The 30-day overall survival was 94%, while the 90-day survival was 88%, see Figure 1. In particular, three neutropenic patients died of septic shock, respectively, 5, 8, and 70 days after viral clearance. The other three patients died from hematological disease progression on days 243, 41, and 98 after the end of treatment. No patient died of SARS-CoV-2 infection.

**Figure 2:**
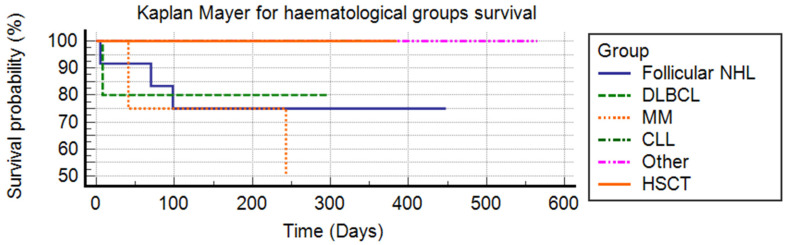
Kaplan–Mayer for hematological group survival (Log-rank test *p* = 0.4497).

No differences in mortality were observed between the different groups related to their hematological condition (log-rank test *p* = 0.4497), see Figure 2.

## 4. Discussion

In our study population, we demonstrated the safety and efficacy of the use of nirmatrelvir/ritonavir and remdesivir in treating immunosuppressed patients with prolonged SARS-CoV-2 infection and disease. No toxicities were observed during antiviral treatment, and no early or late clinical relapse was reported during follow-up. To our knowledge, this is the largest case description currently available.

Even though favorable outcomes were described, both convalescent plasma and monoclonal antibodies (mAbs) were not associated with a combined antiviral regimen given their low clinical efficacy reported [18] and negligible activity against circulating SARS-CoV-2 variants [19].

Early data, mostly case reports, support the off-label use of a combination of SARS-CoV-2 antivirals (remdesivir and nirmatrelvir/ritonavir) to clear the infection in this specific setting. This observation has been supported by symptom recrudescence after 5 days of nirmatrelvir/ritonavir [1] and 10 days of remdesivir treatment, even in a non-immunocompromised population [3]. According to these data, remdesivir has been used as monotherapy for up to 30 days [2] in a hematological setting or in combination with nirmatrelvir/ritonavir for shorter courses [3]. In vitro studies have demonstrated the synergic activity of this antiviral combination against SARS-CoV-2 replication [20].

In our study, differently to previously reported experiences [20,21,22], the follow-up period was longer [18,19,20], and we were able to demonstrate a sustained virological response to antivirals, including, for the 13 (38%) patients who had been subsequently exposed to chemo-immunotherapy.

A short 5-day combination treatment [22] of nirmatrelvir/ritonavir and remdesivir was described with variable outcomes. In Segura Fabrega et al.’s study [23], this short combination treatment was effective, but no follow-up was reported. Similarly to our experience, Pasquini et al. show complete clinical response after 10 days of combination therapy, observing no recurrence during a shorter follow-up period [21,22,23]. Another study [21] combined remdesivir for 10 days with nirmatrelvir/ritonavir for 5 days plus mAbs, with a response rate of 73% and 82%, respectively, on day 30 and day 6. Notably, four patients needed re-treatment due to clinical SARS-CoV-2 infection relapse. These results confirm dual antiviral therapy effectiveness, although administering both antivirals for 10 days appeared to achieve a longer recurrence-free survival rate? Another previous study [24] showed virological clearance through RT-PCR with 5 days of dual combined therapy in 100% of the patients (15/15), but, again, 40% of the subjects experienced SARS-CoV-2 infection recurrence in the following 3 months. In our study, just 23 (68%) of the patients had a negative antigenic test on day 5, suggesting that a therapy course of 5 days may not be enough in an immunosuppressed population.

Moreover, differently from other studies, we prolonged the administration of antivirals up to virological clearance (two consecutive antigenic tests or a negative RT-PCR), which could be a possible reason for such high sustained virological clearance in our case series.

Regarding well-known antiviral side effects, such as asymptomatic bradycardia [21], lactatemia, neutropenia, and liver toxicities [23], we did not observe any of these adverse events with the prolonged course of combination therapy.

B-cell depletion due to anti-CD20 antibody therapy and chemotherapy appeared as one of the most relevant risk factors associated with viral persistence and reactivation.

Moreover, we used covi-FERON^®^ (SD BIOSENSOR, Republic of Korea), an interferon gamma release test, to evaluate the T-cell response to SARS-CoV-2 in a subgroup of 19 patients. Notably, all subjects (19/19) had positive SARS-CoV-2 serology (anti-Spike1-RDB IgG, CMIA) at treatment baseline, but only 7/19 had a positive interferon gamma release assay. Four of the seven patients with a positive T-cell response had previously received multiple doses of rituximab. The scarce viral control, despite the high rate of positive serology, can probably be explained by the necessity of a T-cell response to clear the SARS-CoV-2 infection and to develop immune memory [25]. B-cell depletion, as a result of anti-CD20 treatment, may also impair naïve CD4+ T-cell homeostasis [6].

Hematological subjects treated with anti-CD20 therapy, but with an adequate CD8+ T-cell response, did not suffer from increased mortality compared with other hematological patients, despite almost complete abrogation of SARS-CoV-2-specific antibodies [7]. B-cell depletion due to anti-CD20 antibodies and chemotherapy has been associated with viral persistence and reactivation, but an association with prolonged viral shedding has not been described yet. Whether B-cell depletion alters the SARS-CoV-2-reactive CD8+ T-cell repertoire is an area of ongoing investigation [6].

At the state of the art, it is not clear which patients may benefit from an early dual antiviral therapy according to different risk factors. Dual antiviral treatment may be evaluated for severely immunosuppressed patients, according to the low rate of side effects.

Our study has several limitations: its single-center, retrospective design; small sample size; heterogeneity in immunosuppressive conditions; varying intervals between infection onset and treatment initiation; and incomplete RT-PCR testing at treatment completion.

As no other effective therapies are available for managing protracted SARS-CoV-2 in immunosuppressed patients, further clinical trials are urgently needed to assess the efficacy of and indications for dual antiviral therapy in this population.

## 5. Conclusions

The use of nirmatrelvir/ritonavir and remdesivir was safe and led to complete clinical and virological clearance.

Our findings demonstrate that even patients with protracted SARS-CoV-2 infection can achieve complete viral clearance with dual antiviral therapy, allowing them to promptly undergo chemo-immunotherapy without any recurrence of the infection. This highlights the effectiveness of a timely antiviral strategy in enabling essential oncologic treatments without delay. Furthermore, this study offers a longer follow-up period than any previously published study, providing valuable insights into the safe clinical management and long-term outcomes of vulnerable patients.

## Figures and Tables

**Table 1:** Clinical and biochemical characteristics.

	Follicular NHL	NHL DLBCL	MM	CLL	Other (AML, ALL, Myelofibrosis)	HSCT	KT	Total
Number of patients	12	5	4	3	4	3	3	34
Men %, (n)	50% (6)	100% (5)	100% (4)	100% (3)	25% (1)	0% (0)	67% (2)	62% (21)
Age (y), median (IQR)	69 (65–74)	68 (73–79)	75 (72–77)	76 (72–83)	65 (63–74)	37 (26–43)	54 (53–56)	66 (60–78)
Rituximab cycle (>5), % (n)	100% (12)	80% (4)	25% (1)	0% (0)	0% (0)	0% (0)	0% (0)	50% (17)
Cyclosporin A, % (n)	0% (0)	0% (0)	0% (0)	0% (0)	0% (0)	100% (3)	100% (3)	18% (6)
Last rituximab administration (days from treatment), median (IQR)	112 (45–185)	109 (24–177)	>400	/	/	/	/	/
Previous prophylaxis with Tigaxevimab—Cilgavimab % (n)	25% (3)	40% (2)	0% (0)	0% (0)	0% (0)	0% (0)	0% (0)	15% (5)
Previous antiviral treatments at symptom onset % (n)	50% (6)	80% (4)	50% (2)	0% (0)	50% (2)	67% (2)	67% (2)	53% (18)
Positivity duration (days) median, (IQR)	40 (24–45)	105 (15–205)	16 (2–28)	14 (6–20)	8 (7–10)	23 (15–35)	44 (21–56)	40 (10–34)
Pneumonia %, (n)	83% (10)	40% (2)	75% (3)	67% (2)	75% (3)	67% (2)	67% (2)	71% (24)
Treatment duration, median (IQR)	12 (10–10)	10 (10–10)	15 (10–18)	10 (10–10)	10 (10–10)	10 (10–10)	19 (9–12)	11 (10–10)
Neutropenia	33% (4)	20% (1)	50% (2)	33% (1)	75% (3)	33% (1)	0% (0)	35% (12)
Hypogammaglobulinemia	75% (9)	80% (4)	100% (4)	100% (3)	75% (3)	67% (2)	0% (0)	74% (25)
5th-day negative test (antigen nasal swab) % (n)	58% (7)	100% (5)	25% (1)	67% (2)	100% (4)	67% (2)	67% (2)	68% (23)
10th-day negative nasal swab test % (n)								
Antigen test	58% (7)	100% (5)	50% (2)	100% (3)	100% (4)	100% (3)	100% (3)	80% (27)
Rt—PCR test	58% (7)	100% (5)	50% (2)	67% (2)	100% (4)	100% (3)	33%% (1)	71% (24)
Isolated BAL positivity	33% (4)	/	/	/	/	/	/	12% (4)
Follow-up from last day of treatment, median (IQR)	207 (104–300)	257 (172–373)	169 (72–260)	348 (330–363)	141 (105–175)	254 (98–336)	220 (149–305)	220 (100–316)
Death %, (n)	25% (3)	20% (1)	50% (2)	0% (0)	0% (0)	0% (0)	0% (0)	18% (6)

Abbreviations: NHL: non-Hodgkin’s lymphoma; DLBCL: diffuse large B-cell lymphoma; CLL: chronic lymphocytic leukemia; AML: acute myeloid leukemia; MM: multiple myeloma; ALL: acute lymphoid leukemia; HSCT: hematopoietic stem cell transplant; KT: kidney transplant; IQR: inter quartile range; BAL: bronco alveolar lavage.

## Data Availability

The data supporting the findings of this study are available from the corresponding author upon reasonable request.

## References

[1] Dioverti V., Salto-Alejandre S., Haidar G. (2022). Immunocompromised Patients with Protracted COVID-19: A Review of “Long Persisters”. Curr. Transplant. Rep..

[2] Carlin A.F., Clark A.E., Chaillon A., Garretson A.F., Bray W., Porrachia M., Santos A.T., Rana T.M., Smith D.M. (2022). Virologic and Immunologic Characterization of Coronavirus Disease 2019 Recrudescence After Nirmatrelvir/Ritonavir Treatment. Clin. Infect. Dis..

[3] Martinez M.A., Chen T.Y., Choi H., Hwang M., Navarathna D., Hao L., Gale M., Camus G., Ramirez H.E., Jinadatha C. (2022). Extended Remdesivir Infusion for Persistent Coronavirus Disease 2019 Infection. Open Forum Infect. Dis..

[4] Helleberg M., Niemann C.U., Moestrup K.S., Kirk O., Lebech A.M., Lane C., Lundgren J. (2020). Persistent COVID-19 in an immunocompromised patient temporarily responsive to two courses of remdesivir therapy. J. Infect. Dis..

[5] Lee C.Y., Shah M.K., Hoyos D., Solovyov A., Douglas M., Taur Y., Maslak P., Babady N.E., Greenbaum B., Kamboj M. (2022). Prolonged SARS-CoV-2 Infection in Patients with Lymphoid Malignancies. Cancer Discov..

[6] Aydillo T., Gonzalez-Reiche A.S., Aslam S., van de Guchte A., Khan Z., Obla A., Dutta J., van Bakel H., Aberg J., García-Sastre A. (2020). Shedding of Viable SARS-CoV-2 after Immunosuppressive Therapy for Cancer. N. Engl. J. Med..

[7] Gur I., Giladi A., Isenberg Y.N., Neuberger A., Stern A. (2022). COVID-19 in Patients with Hematologic Malignancies: Clinical Manifestations, Persistence, and Immune Response. Acta Haematol..

[8] Cesaro S., Ljungman P., Mikulska M., Hirsch H.H., von Lilienfeld-Toal M., Cordonnier C., Meylan S., Mehra V., Styczynski J., Marchesi F. (2022). Recommendations for the management of COVID-19 in patients with haematological malignancies or haematopoietic cell transplantation, from the 2021 European Conference on Infections in Leukaemia (ECIL 9). Leukemia.

[9] Nimgaonkar I., Yoke L.H., Roychoudhury P., Flaherty P.W., Oshima M.U., Weixler A., Gauthier J., Greninger A.L., Mielcarek M., Boeckh M. (2024). Outcomes in Hematopoietic Cell Transplant and Chimeric Antigen Receptor T Cell Therapy Recipients with Pre-Cellular Therapy SARS-CoV-2 Infection. Clin. Infect. Dis..

[10] Azzi Y., Bartash R., Scalea J., Loarte-Campos P., Akalin E. (2021). COVID-19 and Solid Organ Transplantation: A Review Article. Transplantation.

[11] Ao G., Wang Y., Qi X., Nasr B., Bao M., Gao M., Sun Y., Xie D. (2021). The association between severe or death COVID-19 and solid organ transplantation: A systematic review and meta-analysis. Transplant. Rev..

[12] Del Bello A., Marion O., Sallusto F., Delas A., Esposito L., Doumerc N., Kamar N. (2021). Kidney transplantation during the COVID-19 pandemic: Potential long-term consequences of an early post-transplant infection. Transpl. Infect. Dis..

[13] Lai Q., Spoletini G., Bianco G., Graceffa D., Agnes S., Rossi M., Lerut J. (2020). SARS-CoV2 and immunosuppression: A double-edged sword. Transplant. Infect. Dis..

[14] Candon S., Guerrot D., Drouot L., Lemoine M., Lebourg L., Hanoy M., Boyer O., Bertrand D. (2021). T cell and antibody responses to SARS-CoV-2: Experience from a French transplantation and hemodialysis center during the COVID-19 pandemic. Am. J. Transplant..

[15] Bakheit A.H., Darwish H., Darwish I.A., Al-Ghusn A.I. (2023). Remdesivir. Profiles Drug Subst. Excip. Relat. Methodol..

[16] Saravolatz L.D., Depcinski S., Sharma M. (2023). Molnupiravir and Nirmatrelvir-Ritonavir: Oral Coronavirus Disease 2019 Antiviral Drugs. Clin. Infect. Dis..

[17] Peracchi F., Merli M., Rogati C., Ravano E., Puoti M., Cairoli R., Travi G. (2023). Dual antiviral therapy in haematological patients with protracted SARS-CoV-2 infection. Br. J. Haematol..

[18] Bloch E.M., Focosi D., Shoham S., Senefeld J., Tobian A.A.R., Baden L.R., Tiberghien P., Sullivan D.J., Cohn C., Dioverti V. (2023). Guidance on the use of convalescent plasma to treat immunocompromised patients with COVID-19. Clin. Infect. Dis..

[19] Tzou P.L., Tao K., Kosakovsky Pond S.L., Shafer R.W. (2022). Coronavirus Resistance Database (CoV-RDB): SARS-CoV-2 susceptibility to monoclonal antibodies, convalescent plasma, and plasma from vaccinated persons. PLoS ONE.

[20] Schultz D.C., Johnson R.M., Ayyanathan K., Miller J., Whig K., Kamalia B., Dittmar M., Weston S., Hammond H.L., Dillen C. (2022). Pyrimidine inhibitors synergize with nucleoside analogues to block SARS-CoV-2. Nature.

[21] Mikulska M., Sepulcri C., Dentone C., Magne F., Balletto E., Baldi F., Labate L., Russo C., Mirabella M., Magnasco L. (2023). Triple Combination Therapy with 2 Antivirals and Monoclonal Antibodies for Persistent or Relapsed Severe Acute Respiratory Syndrome Coronavirus 2 Infection in Immunocompromised Patients. Clin. Infect. Dis..

[22] Pasquini Z., Toschi A., Casadei B., Pellegrini C., D’Abramo A., Vita S., Beccacece A., Bussini L., Chionsini M.C., Dentale N. (2023). Dual combined antiviral treatment with remdesivir and nirmatrelvir/ritonavir in patients with impaired humoral immunity and persistent SARS-CoV-2 infection. Hematol. Oncol..

[23] Fábrega A.S., Catalán I.P., Alfaro I.G., Muñoz S.G., Martí C.R., Lozano N.R., Folgado Escudero S., Villanueva M.V., Gascón Buj A., Torres García M. (2023). Association of nirmatrelvir/ritonavir and remdesivir as treatment for SARS-CoV-2 infection in immunocompromised patients with hematologic malignancies. Series of four cases. Rev. Esp. Quimioter..

[24] Meijer S.E., Halutz O., Adler A., Levytskyi K., Tau L., Dekel M., Cohen-Poradosu R., Katchman E., Shasha D., Ablin J. (2024). Dual anti-viral treatment for persistent COVID-19 in immunocompromised hemato-oncological patients is associated with a favorable prognosis and minor side effects. J. Infect. Chemother..

[25] Naimi A., Yashmi I., Jebeleh R., Imani Mofrad M., Azimian Abhar S., Jannesar Y., Heidary M., Pakzad R. (2022). Comorbidities and mortality rate in COVID-19 patients with hematological malignancies: A systematic review and meta-analysis. J. Clin. Lab. Anal..

